# GABA_A_ Receptor Availability Changes Underlie Symptoms in Isolated Cervical Dystonia

**DOI:** 10.3389/fneur.2018.00188

**Published:** 2018-04-04

**Authors:** Brian D. Berman, Rebecca Tran Pollard, Erika Shelton, Ramesh Karki, Peter M. Smith-Jones, Yubin Miao

**Affiliations:** ^1^Department of Neurology, School of Medicine, University of Colorado Anschutz Medical Campus, Aurora, CO, United States; ^2^Department of Radiology, School of Medicine, University of Colorado Anschutz Medical Campus, Aurora, CO, United States; ^3^Neurology Section, Denver VA Medical Center, Denver, CO, United States; ^4^Department of Psychiatry, School of Medicine, Stony Brook University, Stony Brook, NY, United States

**Keywords:** cervical dystonia, positron emission tomography, GABA receptors, motor cortex, cerebellum

## Abstract

GABA_A_ receptor availability changes within sensorimotor regions have been reported in some isolated forms of dystonia. Whether similar abnormalities underlie symptoms in cervical dystonia is not known. In the present study, a total of 15 cervical dystonia patients and 15 age- and sex-matched controls underwent ^11^C-flumazenil PET/CT scanning. The density of available GABA_A_ receptors was estimated using a Simplified Reference Tissue Model 2. Group differences were evaluated using a two-sample *T*-test, and correlations with dystonia severity, as measured by the Toronto Western Spasmodic Torticollis Rating Scale, and disease duration were evaluated using a regression analysis. Voxel-based analyses revealed increased GABA_A_ availability within the right precentral gyrus in brain motor regions previously associated with head turning and the left parahippocampal gyrus. GABA_A_ availability within the bilateral cerebellum was negatively correlated with dystonia severity, and GABA_A_ availability within the right thalamus and a variety of cerebellar and cortical regions were negatively correlated with disease duration. While GABA_A_ availability changes within primary motor areas could represent a partial compensatory response to loss of inhibition within sensorimotor network, GABAergic signaling impairment within the cerebellum may be a key contributor to dystonia severity.

## Introduction

Cervical dystonia (CD), the most common form of adult-onset focal dystonia (1), is a neurological disorder characterized by involuntary and sustained muscle contractions leading to intermittent or sustained abnormal postures and/or tremor of the neck (2). Although the pathogenesis of CD and other types of focal dystonia remains elusive, a variety of investigative lines support that loss of inhibition plays a key role (3–5).

In a positron emission tomography (PET) using ^11^C-flumazenil, a radiolabeled competitive antagonist to the benzodiazepine binding site on the GABA_A_ receptor, Garibotto et al. reported finding decreased binding was found in sensorimotor cortex, premotor cortex, insula, and anterior cingulate cortex in a cohort of patients with focal, segmental, and generalized dystonia (6). While these findings suggest that GABA signaling dysfunction could be a shared mechanism across dystonia phenotypes, the study was limited by the small number and mixed phenotype of dystonia patients included.

More recently, Gallea et al. reported in a ^11^C-flumazenil PET study that GABA_A_ receptor availability was decreased in the sensorimotor cortex and cerebellum of 18 patients with focal hand dystonia (FHD) compared with 18 healthy controls (HCs) (7). These findings in a more homogenous cohort suggest that altered GABAergic signaling within the sensorimotor network and cerebellum could be an important pathophysiological feature of focal dystonia. Whether a similar pathogenic mechanism underlies CD, however, is not known.

In the present study, we investigated GABA_A_ binding in CD patients and HC, and tested for correlations between GABA_A_ binding and symptom severity and duration. Based on the distribution of findings from the previously published ^11^C-flumazenil PET studies in dystonia (6, 7), we investigated GABA_A_ binding using a whole-brain voxel-based approach and hypothesized that GABA_A_ availability would be altered within sensorimotor regions and the cerebellum. We further hypothesized that decreases in GABA_A_ availability would correlate with dystonia severity.

## Materials and Methods

### Participants

Fifteen CD patients (11F; 63.8 ± 7.8 years) and 15 HC (10F; 64.3 ± 8.9 years) were recruited for the study. There were no significant differences in their age (*p* = 0.86) or sex (*p* = 0.70). All patients had normal neurologic examinations except for their CD symptoms and had predominant dystonia symptoms (as opposed to dystonic tremor). Patients with minimal head jerking and tremor were enrolled only if they had complete resolution of these symptoms when lying down. Severity of dystonia was assessed using the Toronto Western Spasmodic Torticollis Rating Scale (TWSTRS) (8).

Healthy controls taking GABAergic medications were excluded from the study, and patients taking GABAergic medications were tapered off them for a minimum of five half-lives prior to scanning. Patients taking GABAergic medications on a prn basis were asked to not take any for at least 4 weeks prior to scanning. Patients taking any other medications used to treat dystonia had to be stable for at least one month, and those receiving botulinum toxin injections were scanned no sooner than 10 weeks from their last injections. Subjects were excluded if they had cognitive impairment (Montreal Cognitive Assessment score < 26) (9). Patients with dystonia symptoms beginning prior to the age of 18, acquired forms of dystonia, or severe dystonia that could impact the ability to remain still during scanning were excluded. This study was carried out in accordance with the recommendations of the Colorado Multiple Institutional Review Board with written informed consent from all subjects. All subjects gave written informed consent in accordance with the Declaration of Helsinki. This study was approved by the Colorado Multiple Institutional Review Board.

### Experimental Design

All participants underwent scanning on a Philips Gemini 64TF PET/CT imaging system (Philips Medical Systems, The Netherlands). Pads, pillows, Velcro straps, and manipulation of body position were used to limit movement and enable head to be positioned in the most neutral position possible. An initial low-dose CT scan was acquired for attenuation correction and anatomical alignment, followed by a 60-min dynamic PET scan acquired after an intravenous 20 mCi bolus of ^11^C-flumazenil. PET images were acquired as 25 sequential three-dimensional frames (10 s × 30 s, 5 min × 1 min, 10 min × 5 min) of the entire brain (90 slices, 2-mm slice sampling). PET data were reconstructed using an LOR-RAMLA algorithm, tissue correction was applied using the individuals CT scan, and the PET data were smoothed with a 5-mm full width at half maximum Gaussian kernel.

### PET Analysis

PET images were subsequently analyzed using the Statistical NonParametric Mapping (SnPM13) toolbox^1^ implemented in SPM8.^2^ Nonparametric analyses employing locally pooled variance estimates were chosen because they can outperform the comparable Statistical Parametric Mapping approach in a multi-subject PET study with overall low degrees of freedom (10, 11). PET images for each participant were realigned to the mean volume. Any participants who after realignment were found to have mean translation >3 mm or rotation >3° were removed from further analysis.

PET images were then normalized to the standardized SPM8 PET template in Montreal Neurological Institute (MNI) space. GABA_A_ binding was estimated by calculating voxel-by-voxel non-displaceable uptake binding potential (BP) with the *QModeling* toolbox (ver. 1.6.2) implemented in SPM8 using the basis function implementation of the two-step Simplified Reference Tissue Model 2 (12). The input kinetics for the reference tissue were derived from the pons, which has been used in prior ^11^C-flumazenil studies and found to be a reliable reference tissue that is highly correlated with the BP values estimated through arterial sampling (13). The pons was drawn on a high-resolution anatomical MRI image acquired separately for each study participant and then normalized to MNI space. To ensure that systematic differences in the pons time-activity curves did not affect our results, we compared standard uptake values in the pons between HC and CD patients (*p* = 0.938).

### Statistical Analyses

Voxel-wise BP differences were evaluated using an independent sample *T*-test using 5,000 permutations and cluster inference as implemented in SnPM where the default statistic is the maximum intensity *t*-statistic image. Significance for our primary outcome was set at a voxel-level uncorrected *p* < 0.001 threshold at the voxel level followed by cluster-level threshold α < 0.05, corrected for multiple comparisons using *3dClustSim* as implemented in AFNI^3^ (version 16.3.15). Finally, voxel-based linear regression analyses using CD patient BP maps and TWSTRS scores as well as disease duration as covariates (analyses conducted separately) were performed in SnPM to identify regions where GABA_A_ binding correlated with disease severity and duration. Significance for these exploratory outcomes was set at a voxel-level uncorrected *p* < 0.005 followed by cluster threshold of 100 contiguous voxels to limit type-I error. Scatter plots generated with extracted mean BP values were inspected to verify significant correlations were not being driven by outliers.

## Results

### Participants

PET data from four CD patients and two HC were removed from analysis due to excess movement. There were no significant differences in administered ^11^C-flumazenil (20.3 ± 0.9 mCi vs. 20.0 ± 0.5 mCi; *p* = 0.39), as well as no significant differences in age (63.0 ± 8.0 vs. 65.3 ± 8.5; *p* = 0.52) or sex (9F:2M vs. 9F:4M; *p* = 0.50) between the remaining CD patients and HC. Clinical characteristics of the CD patients included in the final analysis are shown in Table 1. Five of the patients had right torticollis, five had left torticollis, and three had minimal dystonic head tremor. Three of the 11 patients were taking one low-dosed GABAergic medication regularly and were weaned off these medications prior to scanning.

**Table 1:** Clinical characteristics of cervical dystonia subjects included in final ^11^C-flumazenil positron emission tomography (PET) analysis.

Subject	Disease duration, years	TWSTRS	Dystonia characteristics	GABAergic medication	BoNT
1	8	23.75	Lt torticollis, Rt laterocollis	N	N
2	47	17.5	Lt torticollis, Lt laterocollis, Lt shoulder elevation, tremor	N	Y
3	11	10	Lt torticollis	Clonazepam prn	Y
4	23	28	Lt torticolls, retrocollis	N	Y
5	7	22.75	Rt torticollis, anterocollis	Zolpidem prn	N
6	14	28	Rt torticollis, anterocollis	N	Y
7	47	19	Rt torticollis, anterocollis	N	Y
8	30	16.25	Rt torticollis, Rt laterocollis, Rt shoulder elevation, tremor	Clonazepam	Y
9	19	11	Rt torticollis, Lt laterocollis	Clonazepam	N
10	15	13.5	Lt torticollis	N	Y
11	13	13.5	Rt torticollis, Rt shoulder elevation, tremor	Primidone	N

*Patients with tremor had predominant dystonia symptoms and only minimal tremor on exam that resolved with lying down and was not expected to interfere with PET acquisition*.

*BoNT, botulinum toxin; Lt, left; Rt, right; TWSTRS, Toronto Western Spasmodic Torticollis Rating Scale*.

### ^11^C-flumazenil BP: CD Patients vs. HC

**Figure 1:**
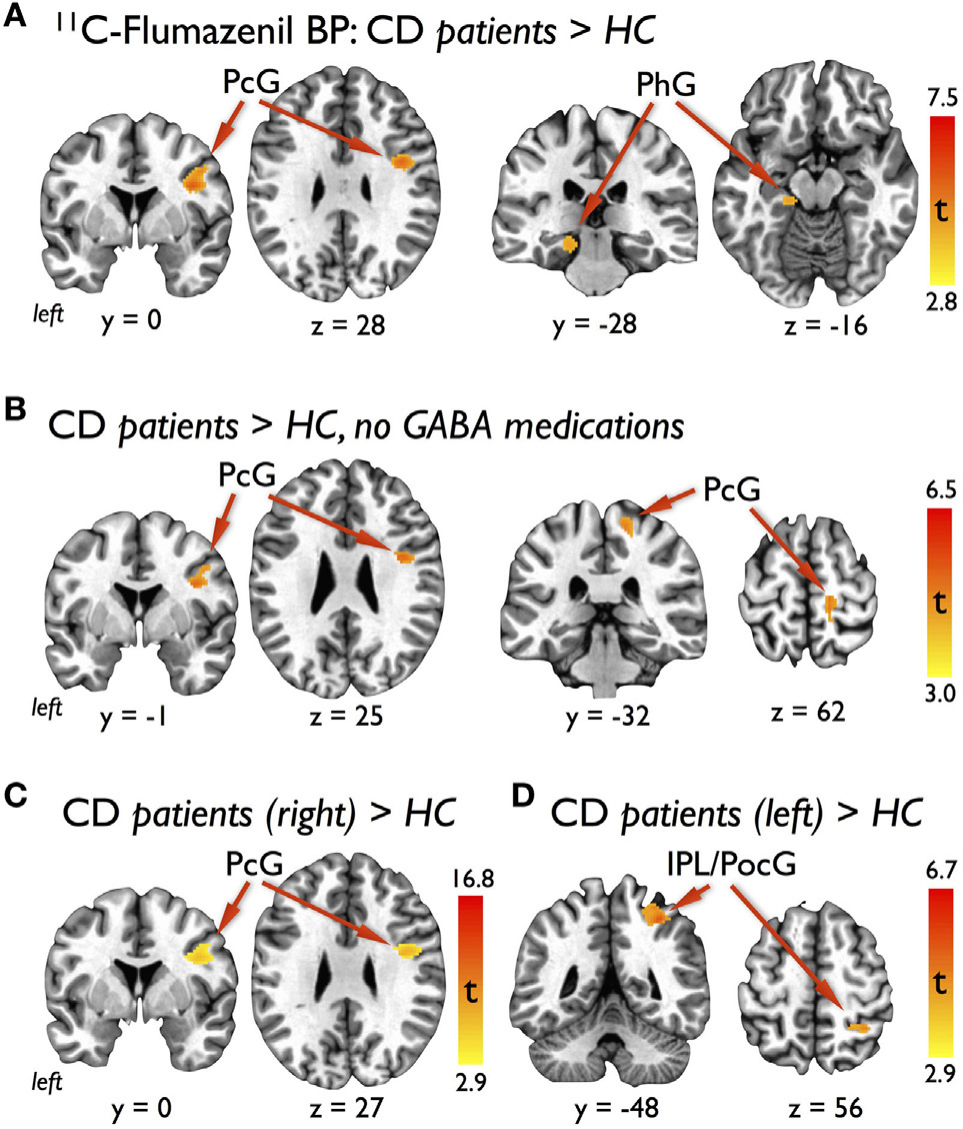
Results of voxel-based analyses showing **(A)** significant clusters in the right lateral precentral gyrus (PcG) and left parahippocampal gyrus (PhG) with increased ^11^C-flumazenil binding potential (BP) in cervical dystonia (CD) patients compared with healthy controls (HCs), **(B)** significant clusters in the right lateral and medial PcG with increased BP in the subgroup of CD patients not taking GABAergic medications compared with HC, **(C)** significant cluster in the right lateral PcG with increased BP in the subgroup of CD patients with right torticollis compared with HC, and **(D)** significant cluster in the right inferior parietal lobe (IPL) and postcentral gyrus (PocG) with increased BP in the subgroup of CD patients with left torticollis compared with HC. Voxel-based findings for the primary outcome **(A)** were FWE corrected for multiple corrections at an individual voxel statistical threshold of *p* < 0.001 with a cluster threshold of at least 102 contiguous voxels for overall α < 0.05. Significance for the subgroup analyses **(B–D)** were defined as an individual voxel statistical threshold of *p* < 0.005 with a cluster threshold of 100 contiguous voxels.

Compared with HC, CD patients had significantly increased ^11^C-flumazenil BP in the right precentral gyrus and left parahippocampal gyrus (Table 2; Figure 1A). There were no other regions of significant increases or decreases in BP found. To verify that the three patients weaned off GABAergic medications did not influence our results, a separate voxel-based independent sample two-tailed *T*-test was run using only the eight CD patients not taking these medications on regular basis. Due to the reduced degrees of freedom, a less-strict voxel-level uncorrected *p* < 0.005 significance threshold followed by a cluster threshold of 100 contiguous voxels was used for this comparison. A significantly increased BP was still found in the right precentral gyrus, along with an additional cluster in the right precentral gyrus that was more medial and dorsal (Table 2; Figure 1B).

**Table 2:** Anatomical localization of significant clusters identified in the voxel-based analyses.

Contrast/correlation	Brain region	MNI Coordinates			Cluster size (voxels)	Peak *t*-value
		*X*	*Y*	*Z*		
CD > HC	Rt precentral gyrus	40	0	26	217	5.65
	Lt parahippocampal gyrus	−18	−28	−16	114	3.72

CD > HC*	Rt precentral gyrus	38	0	26	109	4.59
	Rt precentral/postcentral gyrus	16	−26	60	100	4.23

CD > HC^R^	Rt precentral gyrus	38	−4	26	181	9.76

CD > HC^L^	Rt inferior/superior parietal lobe/postcentral gyrus	34	−48	52	110	5.12

Severity	Lt cerebellum (lobule VIIIa)	−32	−46	-58	169	−9.24
	Lt cerebellum (crus 2)	−42	−70	−46	152	−7.32
	Rt cerebellum (crus 1/2)	22	−74	−34	126	−7.40
	Lt cerebellum (crus 1)	−28	−66	−36	111	−7.38

Duration	Cerebellar vermis	4	−36	−10	159	−9.56
	Lt cerebellum (crus 2)	−6	−92	−36	151	−8.97
	Rt temporal pole	48	6	−20	124	−9.12
	Rt thalamus	18	−30	6	102	−7.22
	Lt cuneus	−12	−68	24	102	−8.18
	Lt olfactory/anterior cingulate cortex, caudate nucleus	−2	14	−12	101	−9.61

Results from voxel-based comparisons of GABA_A_ receptor availability between cervical dystonia patients (CD) and healthy controls (HCs) and from voxel-wise correlation between GABA_A_ receptor availability and disease severity as measured by Toronto Western Spasmodic Torticollis Rating Scale (TWSTRS) and disease duration. *Reflects voxel-based comparison after removal of three patients weaned off GABAergic medications; ^R^reflects voxel-based comparison between CD patients with right turning torticollis and HC, and Lreflects voxel-based comparison between CD patients with left turning torticollis and HC. Main contrast between CD and HC is FWE corrected for multiple comparisons at individual voxel threshold *p* < 0.001 and *k* > 102. All other voxel-based comparisons at individual voxel threshold *p* < 0.005 with cluster size threshold *k* = 100.

*Lt, left; MNI, Montreal Neurological Institute; Rt, right*.

To explore whether our GABA_A_ binding results were influenced by the direction of head turning, we conducted two additional independent sample two-tailed *T*-tests with CD patients separated into those who had head turning to the right (*N* = 6) and those who had head turning to the left (*N* = 5). Using a similar voxel-level uncorrected *p* < 0.005 significance threshold and cluster threshold of 100 contiguous voxels for these comparisons, we found that the CD patients with right head turning showed persistence of the significant cluster of increased ^11^C-flumazenil BP in the right precentral gyrus (Table 2; Figure 1C) and that the CD patients with left head turning had a single cluster of increased BP that overlapped the postcentral gyrus and inferior and posterior parietal lobes (Table 2; Figure 1D). No regions of decreased BP were found for either comparison.

### Correlations Between ^11^C-flumazenil BP and Clinical Features

**Figure 2:**
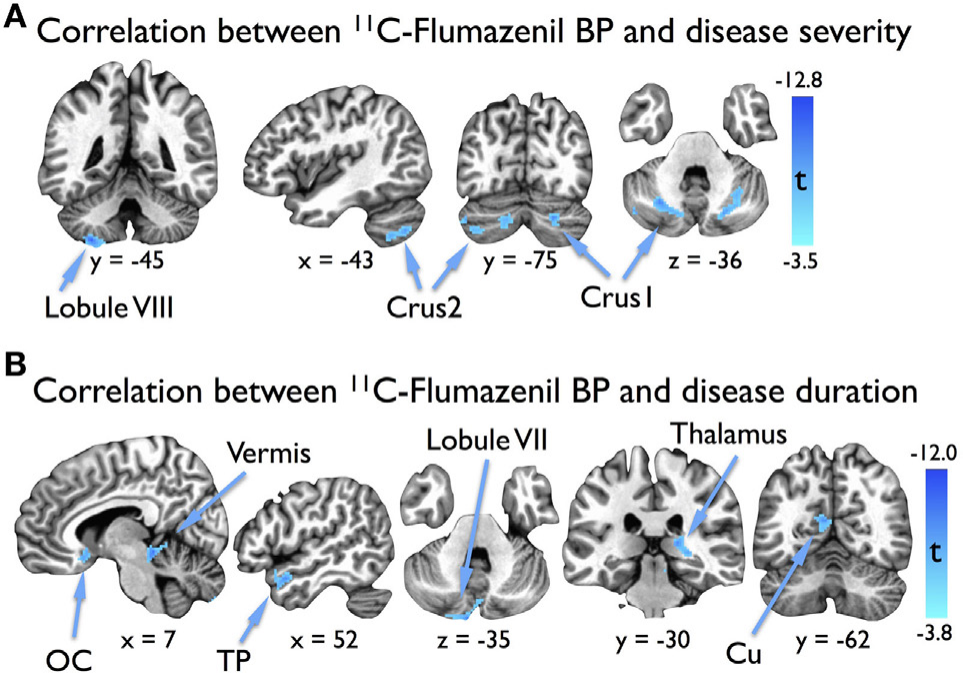
Results of voxel-based regression analyses showing **(A)** significant clusters in the bilateral cerebellar hemispheres where ^11^C-flumazenil binding potential (BP) in cervical dystonia (CD) patients correlated with dystonia severity as measured by the Toronto Western Spasmodic Torticollis Rating Scale (TWSTRS), and **(B)** significant clusters where ^11^C-flumazenil BP in CD patients correlated with disease duration. Significance was defined as an individual voxel statistical threshold of *p* < 0.005 with a cluster threshold of 100 contiguous voxels. Images are displayed such that left is anatomical left. Abbreviations: Cu, cuneus; OC, olfactory cortex; TP, temporal pole.

In our regression analysis, BP showed significant negative correlations with TWSTRS in the bilateral cerebellar hemispheres (Table 2; Figure 2A). Significant negative correlations with disease duration were found in the cerebellar vermis, left cerebellar hemisphere, right thalamus, right temporal pole, and left cuneus, as well as in a cluster that overlapped the left olfactory cortex, anterior cingulate cortex, and caudate nucleus (Table 2; Figure 2B).

## Discussion

Using ^11^C-flumazenil PET/CT, we found increased GABA_A_ binding within a lateral portion of the right precentral gyrus of CD patients compared with HC. In a *post hoc* analysis after three CD patients with recent exposure to low-dose GABAergic medications were removed, we found an additional region of increased GABA_A_ binding in a more medial portion of the right precentral gyrus. Supporting the relevance of these findings to CD, the two primary motor areas are anatomically consistent with the presence of a dual representation of the neck in the motor homunculus as recently demonstrated in a recent functional MRI study of healthy individuals performing a head rotation task (14). Increased GABA_A_ binding was also found in the left parahippocampal gyrus in CD patients, but the relevance of this finding to CD is less clear. Increases in the volume of this gray matter cortical region (15), as well as activation differences during a motor task (16) and visuospatial task (17), however, have previously been reported in CD patients when compared with controls, suggesting that the region may play some role in the disorder.

Gallea et al. found regions of increased GABA_A_ availability in the bilateral inferior frontal gyrus of FHD patients (7). The increased availability in their study was interpreted as potentially representing a compensatory response to dysfunction within the sensorimotor network of FHD patients. Similarly, the increased GABA_A_ availability in the primary motor cortex of our CD patients may stem from adaptive or compensatory mechanisms. For example, increased GABA_A_ availability could be a response to reduced GABAergic inputs to pyramidal neurons, which normally inhibit their neighboring glutamatergic pyramidal neurons (18), as has been reported to occur in patients with schizophrenia (19). Alternatively, increased GABA_A_ availability might derive from over activity of glutamatergic efferents from the ventral lateral nucleus of the thalamus, which serves as the primary pathway involved in the transference of cerebellar input to the primary motor cortex (20). Irrespective of the precise mechanism involved, our findings in conjunction with those recently reported in FHD support that focal dystonia patients retain an ability to partially compensate for GABAergic signaling deficits, but that the location of adaptive changes could depend on body region affected or task-specificity of dystonia.

Of note, the first reported study of ^11^C-flumazenil PET in dystonia reported only decreased GABA_A_ availability in sensorimotor regions and no areas of increased GABA_A_ availability (6). The lack of increased GABA_A_ availability reported in this study by Garibotto et al. could stem from the fact that only two dystonia patients in their study had isolated focal dystonia, one with CD and one with FHD, with two patients having segmental dystonia and the remaining 10 having generalized dystonia (6). As such, the spread and generalization of dystonia may represent a diminished or absent ability to compensate for GABAergic signaling deficits.

The cerebellum has increasingly been recognized as contributing to the pathophysiology of dystonia (21, 22). In our study, we found regions within the cerebellum showing significant correlations between decreasing GABA_A_ availability and worsening motor severity in patients. Negative correlations between GABA_A_ availability and dystonia severity in the left inferior posterior cerebellar lobe (Lobule VIII), which contains the representation of the sensorimotor cerebellum (23), and lateral cerebellar hemispheres, which send efferents through the dentate nucleus that join inputs from the basal ganglia in the ventral anterior and ventral lateral nuclei of the thalamus and play a role in motor planning, initiation, and coordination (24), raises the possibility that dysfunction within cerebellar circuits influences the severity of dystonic symptoms in CD. Although Gallea et al. found regions of decreased GABA_A_ availability within the vermis and superior posterior lobe in FHD patients, their patient population precluded them from testing for a correlation between GABA_A_ availability and dystonia severity (7). In a study of nine dystonia patients (seven with CD) who had direct local field potentials recorded from their pallida, however, pallido-cerebellar coupling in the alpha band range (7–13 Hz) was negatively correlated with TWSTRS scores (25). In combination, these findings support the presence of impaired GABAergic signaling within the cerebellum and that this impairment may disrupt pallido-cerebellar coupling.

In our study, we found several dispersed clusters showing significant negative correlations between GABA_A_ availability and disease duration in CD patients. Presently, no comparable study in focal dystonia exists to assess the robustness of these findings. While Gallea et al. tested for correlations between disease duration and both cerebral blood flow and gray matter volumes in FHD patients, no voxel-wise correlation analysis between disease duration and GABA_A_ binding was reported (7). Nevertheless, they did report a negative correlation between cerebral blood flow in the inferior frontal cortex and disease duration. A variety of investigations have also reported findings that support that neurobiological changes in CD are associated disease duration. For example, microstructural alterations within a number of brain areas spanning the frontal, temporal, parietal lobe, and occipital lobes have been reported to correlate with disease duration (15), and longer disease duration may reduce the therapeutic effect of deep brain stimulation of the globus pallidus internus in CD (26). In patients with craniocervical dystonia, gray matter volume declines in the frontal lobe, parietal lobe, and cerebellar vermis and hemispheres have also been reported to correlate with disease duration (27). These findings combined with our results suggest that GABA_A_ availability may decrease in several of specific brain regions with disease duration in CD.

An intriguing finding here is the laterality of our results in the primary motor cortex. Our CD population had a mix of dystonia-induced neck postures (Table 1), arguing against a specific set of the muscles contributing to the asymmetric findings. Additionally, reducing our individual voxel threshold from *p* = 0.001 to *p* = 0.05 (and lower) did not reveal increased GABA_A_ binding in the left precentral gyrus (data not shown). Separating our CD cohort into those with right and left turning torticollis further revealed that the increase in GABA_A_ binding in the right precentral gyrus was more strongly associated with those CD patients with right torticollis. This again may support that the increased GABA_A_ availability is a compensatory response, possibly in response to a loss of inhibition affecting the left hemisphere and resulting in excessive firing of cervical musculature turning the head to the right. CD patients with left torticollis, however, also showed a region of increased GABA_A_ binding present on the right, though this was more posterior and in the region of the postcentral gyrus. Increased GABA_A_ availability within somatosensory cortex could stem from impaired sensorimotor integration as has been described in CD (28), but it is unclear why the finding was evident only in CD patients with left torticollis. Still, the persistence of right-sided findings in patients with both right and left turning torticollis suggests that CD may be associated with an underlying asymmetric pathophysiology.

Further evidence for an asymmetry in the pathophysiology underlying CD comes from recent microelectrode recording and imaging studies. In one study of 13 patients with CD undergoing deep brain stimulation surgery, asymmetric outflow from the globus pallidus internus was reported (29). In a diffusion tensor imaging study of 12 CD patients, tractography changes between the pallidum and brainstem were affected in opposite ways in the two hemispheres (30). Taken together, these findings support that CD could arise from an underlying asymmetry pathophysiology. Negative correlations between GABA_A_ binding and dystonia severity, however, were seen in bilateral cerebellar regions supporting presence of a more symmetric alteration in inhibitory signaling within the cerebellum is present in CD. Further investigation with larger sample sizes will be needed to determine whether pathophysiological differences truly exist between those with right and left torticollis and whether an asymmetric pathophysiology underlies CD or other types of isolated dystonia.

Although our overall enrollment is reasonable for a PET study of this nature, one of the limitations of our study is the small sample size. Due to the dynamic ^11^C-flumazenil PET scan acquisition taking 60 min and our studying of dystonia that affects neck muscles, we opted to apply strict motion criteria in favor of higher quality data resulting in removal of data from six participants. Our lower number of subjects, however, resulted in a relatively high statistical threshold (*t* = 2.8) for our two-group contrast, potentially reducing the number of significant findings we found and limiting the ability to directly compare our findings to those recently reported by Gallea et al. where all 36 participants were included in the final analysis (7). Another potential limitation in our study is that three of the CD patients scanned were taking medications that potentially could interfere with GABA_A_ availability. In each case, however, patients were on low doses and tapered off well before their scans. Additionally, our primary finding persisted in an exploratory analysis after removal of these three CD patients. And finally, if it were to cause an effect on our findings, the chronic use of benzodiazepines would be expected to likely have a downregulation effect on the number or availability of GABA_A_ receptors rather than an upregulation effect (31).

In summary, we found abnormally increased GABA_A_ availability in the right precentral gyrus of CD patients in a region of motor cortex associated with activating the muscles involved in head turning, along with negative correlations between dystonia severity and GABA_A_ availability in the cerebellar hemispheres, as well as negative correlations between disease duration and GABA_A_ availability in the right thalamus, cerebellar vermis, left cerebellar hemisphere, and other cortical regions. These findings support the presence of altered GABAergic signaling in CD and raise the possibility that reduced GABAergic inhibition by the cerebellum may be a driving force of dystonia severity.

## Ethics Statement

This study was carried out in accordance with the recommendations of the Colorado Multiple Institutional Review Board. All subjects gave written informed consent in accordance with the Declaration of Helsinki. This study was approved by the Colorado Multiple Institutional Review Board.

## Author Contributions

BB was responsible for research project conception, organization, and execution, contributed to the statistical analysis design and execution, wrote the first draft manuscript, and reviewed and edited the final draft of the manuscript. RP contributed to research project organization and execution, statistical analysis execution, and review and critique of the manuscript. ES contributed to research project organization and execution, assisted with statistical analysis execution, and review and critique of the manuscript. RK contributed to research project organization and execution, and review and critique of the manuscript. PS-J contributed to the research project conception, organization, and execution, as well as review and critique of the manuscript. YM contributed to research project execution, statistical analysis review, and review and critique of the manuscript.

## Conflict of Interest Statement

The authors declare that the research was conducted in the absence of any commercial or financial relationships that could be construed as a potential conflict of interest.

## Footnotes

^1^http://warwich.ac.uk/snpm.

^2^http://www.fil.ion.ucl.ac.uk/spm.

^3^https://afni.nimh.nih.gov.
